# Maternal blood pressure associates with placental DNA methylation both directly and through alterations in cell-type composition

**DOI:** 10.1186/s12916-022-02610-y

**Published:** 2022-10-20

**Authors:** Lucile Broséus, Daniel Vaiman, Jörg Tost, Camino Ruano San Martin, Milan Jacobi, Joel D. Schwartz, Rémi Béranger, Rémy Slama, Barbara Heude, Johanna Lepeule

**Affiliations:** 1 University Grenoble Alpes, INSERM, Team of Environmental Epidemiology Applied to Development and Respiratory Health, Institute for Advanced Biosciences (IAB), Grenoble, France; 2 From Gametes to Birth, Institut Cochin, U1016 INSERM, UMR 8104 CNRS, Paris-Descartes University, Paris, France; 3 Laboratory for Epigenetics and Environment, Centre National de Recherche en Génomique Humaine, CEA – Institut de Biologie François Jacob, University Paris Saclay, Evry, France; 4 Department of Environmental Health, Harvard T.H. Chan School of Public Health, Boston, MA USA; 5 Univ. Rennes, CHU Rennes, INSERM, EHESP, IRSET (Institut de recherche en santé, environnement et travail), UMR 1085, Rennes, France; 6 Univ. Paris, Centre for Research in Epidemiology and Statistics (CRESS), INSERM, INRAE, Paris, France

**Keywords:** Placenta, DNA methylation, Blood pressure, Pregnancy, Cell-type heterogeneity, Mesenchymal stromal cells, Epigenome-wide association study

## Abstract

**Background:**

Maternal blood pressure levels reflect cardiovascular adaptation to pregnancy and proper maternal-fetal exchanges through the placenta and are very sensitive to numerous environmental stressors. Maternal hypertension during pregnancy has been associated with impaired placental functions and with an increased risk for children to suffer from cardiovascular and respiratory diseases later on. Investigating changes in placental DNA methylation levels and cell-type composition in association with maternal blood pressure could help elucidate its relationships with placental and fetal development.

**Methods:**

Taking advantage of a large cohort of 666 participants, we investigated the association between epigenome-wide DNA methylation patterns in the placenta, measured using the Infinium HumanMethylation450 BeadChip, placental cell-type composition, estimated in silico, and repeated measurements of maternal steady and pulsatile blood pressure indicators during pregnancy.

**Results:**

At the site-specific level, no significant association was found between maternal blood pressure and DNA methylation levels after correction for multiple testing (false discovery rate < 0.05), but 5 out of 24 previously found CpG associations were replicated (*p*-value < 0.05). At the regional level, our analyses highlighted 64 differentially methylated regions significantly associated with at least one blood pressure component, including 35 regions associated with mean arterial pressure levels during late pregnancy. These regions were found enriched for genes implicated in lung development and diseases. Further mediation analyses show that a significant part of the association between steady blood pressure—but not pulsatile pressure—and placental methylation can be explained by alterations in placental cell-type composition. In particular, elevated blood pressure levels are associated with a decrease in the ratio between mesenchymal stromal cells and syncytiotrophoblasts, even in the absence of preeclampsia.

**Conclusions:**

This study provides the first evidence that the association between maternal steady blood pressure during pregnancy and placental DNA methylation is both direct and partly explained by changes in cell-type composition. These results could hint at molecular mechanisms linking maternal hypertension to lung development and early origins of childhood respiratory problems and at the importance of controlling maternal blood pressure during pregnancy.

**Supplementary Information:**

The online version contains supplementary material available at 10.1186/s12916-022-02610-y.

## Background

During pregnancy, placental proper development and functions are crucial for both maternal and fetal health. The placenta is involved in supplying nutrients and oxygen to the fetus, removing gas and waste products, and regulating fetal growth through the production of growth hormones [1]. These exchanges are made through placental microcirculation and are influenced by maternal blood flow. Prevalent hypertensive disorders of pregnancy (HDP), such as preeclampsia, are believed to originate from placental dysfunction [2]. But a growing body of evidence suggests that maternal blood flow could also be a primary determinant of the onset of pregnancy disorders [3]. Pre- and early pregnancy maternal blood pressure (BP) levels were indeed found to be predictive of later placental malperfusion and women with chronic hypertension are at higher risk for pregnancy complications [4–6]. Moreover, maternal BP during pregnancy and gestational hypertension are associated with impaired placental development, adverse pregnancy outcomes, low birth weight and size, and an increased risk for children to suffer from cardiovascular diseases and respiratory problems, but the mechanisms underpinning these relationships are still poorly understood [6–8].

Accumulating evidence suggests that changes in placental DNA methylation (DNAm) may provide a readout of placental state and pregnancy history and a means to decipher the underlying mechanisms of action by which maternal health conditions during pregnancy affect fetal and later-life health [9, 10]. Furthermore, alterations in DNAm signals may originate from *direct* modifications, which affect all cell types, but also from *indirect* variations mediated by changes in cell-type composition [11]. The importance of accounting for cell-type heterogeneity in epigenome-wide association studies (EWAS) has been extensively discussed, yet methodological recommendations to address this question are scarce and often reduce to removing confounding cell-mixture effects through regression models [12, 13]. This approach might not be optimal when changes in cell-type distribution are a pathway to explain the association between an exposure and DNAm. In that case, explicitly distinguishing between shifts in cell-type proportions from direct changes in DNAm could provide valuable insights into the biological mechanisms of placental cellular differentiation and development throughout pregnancy [14]. A few EWAS relating maternal BP to DNAm were performed, with most being case-control studies focused on HDP in which DNAm had been measured in cord blood [15]. Only one study specifically interrogated the linear dose-effect relationship between maternal systolic blood pressure (SBP) and diastolic blood pressure (DBP) levels and placental DNAm [16]. However, these studies included a smaller number of participants and did not estimate the potential effect of cell mixture, and findings did not consider mean arterial pressure (MAP) nor pulse pressure (PP), which are relevant indicators to consider during pregnancy and independent predictors of the risk of cardiovascular diseases [17–19].

We hypothesized that maternal BP could influence placental DNAm, and taking advantage of recent experimental advances to estimate the proportion of six reference cell types in human term placenta [20], we investigated the mediating role played by cell-type distribution. We examined the association between placental DNAm; three steady hemodynamic parameters, SBP, DBP, and MAP; and one pulsatile parameter, PP, in the large French EDEN cohort [21]. To our knowledge, this is the first study of the association of maternal steady and pulsatile components of blood pressure to placental DNAm and of the extent to which it operates through changes in placental cell-type composition.

## Methods

### Study design

#### Population

The present study relies on 668 mother-child pairs from the French cohort EDEN, who were recruited between 2003 and 2006 [21]. Recruitment of pregnant women at the Nancy and Poitiers University hospitals took place before their 24th week of gestation. Exclusion criteria were as follows: maternal diabetes before pregnancy, multiple fetuses, intention to deliver outside the university hospital or to move out of the study region within the next 3 years, and inability to speak French. Out of the 2002 enrolled participants, 1301 had placental samples collected and placental DNAm was assessed for 668 of them. Out of these, 666 women had at least one abstracted blood pressure measurement and available information on considered covariates (Table 1). Gestational age was estimated using the date of the last menstrual periods.

**Table 1:** Characteristics of EDEN participants with available blood pressure and DNA methylation measurements for each considered time window. BMI, body mass index; GDM, gestational diabetes mellitus; HDP, hypertensive disorder in pregnancy; SBP, systolic blood pressure; DBP, diastolic blood pressure; MAP, mean arterial pressure; PP, pulse pressure; early pregnancy: 0–27 weeks of gestation; late pregnancy: >27 weeks of gestation to delivery

	Early pregnancy *N* = 665	Late pregnancy *N* = 653	Pregnancy *N* = 666
Center of recruitment:			
Nancy	380 (57.1%)	378 (57.9%)	381 (57.2%)
Poitiers	285 (42.9%)	275 (42.1%)	285 (42.8%)
Maternal age ( years )	29.0 [25.0; 32.0]	29.0 [25.0; 32.0]	29.0 [25.0; 32.0]
Maternal parity:			
>0	365 (54.9%)	360 (55.1%)	366 (55.0%)
0	299 (45.0%)	292 (44.7%)	299 (44.9%)
Missing	1 (0.15%)	1 (0.15%)	1 (0.15%)
Child sex:			
Female	317 (47.7%)	314 (48.1%)	318 (47.7%)
Male	348 (52.3%)	339 (51.9%)	348 (52.3%)
maternal pre-gravid BMI (kg/m^2^):			
normal	454 (68.3%)	445 (68.1%)	455 (68.3%)
obesity	40 (6.02%)	39 (5.97%)	40 (6.01%)
overweight	112 (16.8%)	110 (16.8%)	112 (16.8%)
underweight	59 (8.87%)	59 (9.04%)	59 (8.86%)
Maternal education attainment (number of years after high school):			
>2 years	420 (63.2%)	415 (63.6%)	421 (63.2%)
0–2 years	113 (17.0%)	110 (16.8%)	113 (17.0%)
0 years	132 (19.8%)	128 (19.6%)	132 (19.8%)
Maternal smoking status:			
Continued smoker	207 (31.1%)	202 (30.9%)	207 (31.1%)
Former smoker	69 (10.4%)	68 (10.4%)	69 (10.4%)
Non-smoker	380 (57.1%)	374 (57.3%)	381 (57.2%)
Missing	9 (1.35%)	9 (1.38%)	9 (1.35%)
Gestational duration (weeks)	40.0 [38.9; 40.9]	40.0 [39.0; 40.9]	40.0 [38.9; 40.9]
Preterm birth:			
No	633 (95.2%)	625 (95.7%)	634 (95.2%)
Yes	32 (4.81%)	28 (4.29%)	32 (4.80%)
Mode of delivery:			
Vaginal	575 (86.5%)	566 (86.7%)	575 (86.3%)
C-section	89 (13.4%)	86 (13.2%)	90 (13.5%)
Missing	1 (0.15%)	1 (0.15%)	1 (0.15%)
GDM:			
No	623 (93.7%)	611 (93.6%)	624 (93.7%)
Yes	42 (6.32%)	42 (6.43%)	42 (6.31%)
HDP:			
Gestational hypertension	17 (2.56%)	17 (2.60%)	17 (2.55%)
Normotensive	630 (94.7%)	619 (94.8%)	631 (94.7%)
Preeclampsia	17 (2.56%)	17 (2.60%)	17 (2.55%)
Missing	1 (0.15%)	0 (0.00%)	1 (0.15%)
BP indicators (original values in mmHg):			
SBP	115 [109; 121]	116 [110; 123]	115 [110; 122]
DBP	64.4 [60.7; 68.4]	65.5 [60.3; 70.0]	64.9 [61.4; 69.2]
MAP	81.1 [77.6; 85.3]	82.5 [77.8; 87.0]	81.6 [78.0; 85.9]
PP	50.0 [45.8; 54.4]	50.0 [45.0; 56.7]	50.2 [46.2; 54.5]
Number of measurements	5.00 [4.00; 6.00]	3.00 [3.00; 4.00]	9.00 [7.25; 10.0]

#### Blood pressure (BP)

SBP and DBP measurements in millimeters of mercury (mmHg) were abstracted from normal follow-up measurements during pregnancy and specific study measurements scheduled at 24 to 28 weeks of gestation. Based on clinical expertise and previous studies, we used upper thresholds of > 260 mmHg for SBP and > 200 mmHg for DBP for determining the plausibility of BP measures [22, 23]. There were no such outlying measurements in our data. Participants having both gestational hypertension and proteinuria according to their obstetric history form were classified as preeclamptic (PE).

#### Measurement of placental DNA methylation

At delivery, placental tissue from the fetal side was sampled at one site by the specifically trained midwives of the study using the following standardized procedure. Samples of around 5 mm^3^ were collected a few centimeters from the insertion of the cord under the chorio-amniotic membrane, washed in a saline solution, and immediately frozen at −80°C. The protocol was similar for both centers and all modes of delivery. DNA was extracted using the QIAsymphony instrument (Qiagen, Germany) [24]. The DNA samples were plated onto nine plates including 64 chips, analyzed in 4 batches, and allocated so that the ratios for sex (boy/girl) and recruitment center (Poitiers/Nancy) were balanced for each chip. Whole-genome DNAm was measured using the Infinium HumanMethylation450 BeadChip. Raw intensities of fluorescent signals were extracted using the GenomeStudio® software (v2011.1. Illumina), processed with Chip Analysis Methylation Pipeline (ChAMP), and the DNAm level of each CpG was calculated as the ratio of the intensity of fluorescent signals of the methylated alleles over the sum of methylated and unmethylated alleles (beta-value) [25]. All samples passed initial quality control and had on average > 98% of valid data points (detection *p*-value < 0.01). Potential sex mix-ups were investigated by comparing placental DNAm-based sex prediction to the annotated sex, which led to exclude one sample from the subsequent analyses. Beta-values were then normalized using the Beta Mixture Quantile (BMIQ) normalization [26]. To reduce the influence of outliers, within each probe, methylation values above the 75th percentile + three interquartile ranges (IQRs) or below the 25th percentile − three IQRs were removed (in total 0.36% of methylation values in the whole dataset). CpG sites at a distance ≤ 2 bp from SNPs with minor allele frequency > 0.05 and chromosomes X and Y were removed using the R package *DMRcate* [27]. As recently noticed, many positive calls in EWAS may be spurious detections caused by cross-reactive probes [28]. Cross-hybridized probes identified in previous studies were thus filtered out using the R package *maxprobes* (https://github.com/markgene/maxprobes) [29, 30]. 367,865 CpG sites remained after quality control and the successive filtering steps (Additional file 1: Figure S1). For each individual, bisulfite pyrosequencing was used to measure the global methylation levels of repetitive *Alu* elements (*Alu*) and long interspersed nucleotide elements 1 (*LINE-1*) [31].

### Statistical analyses

**Fig. 1:**
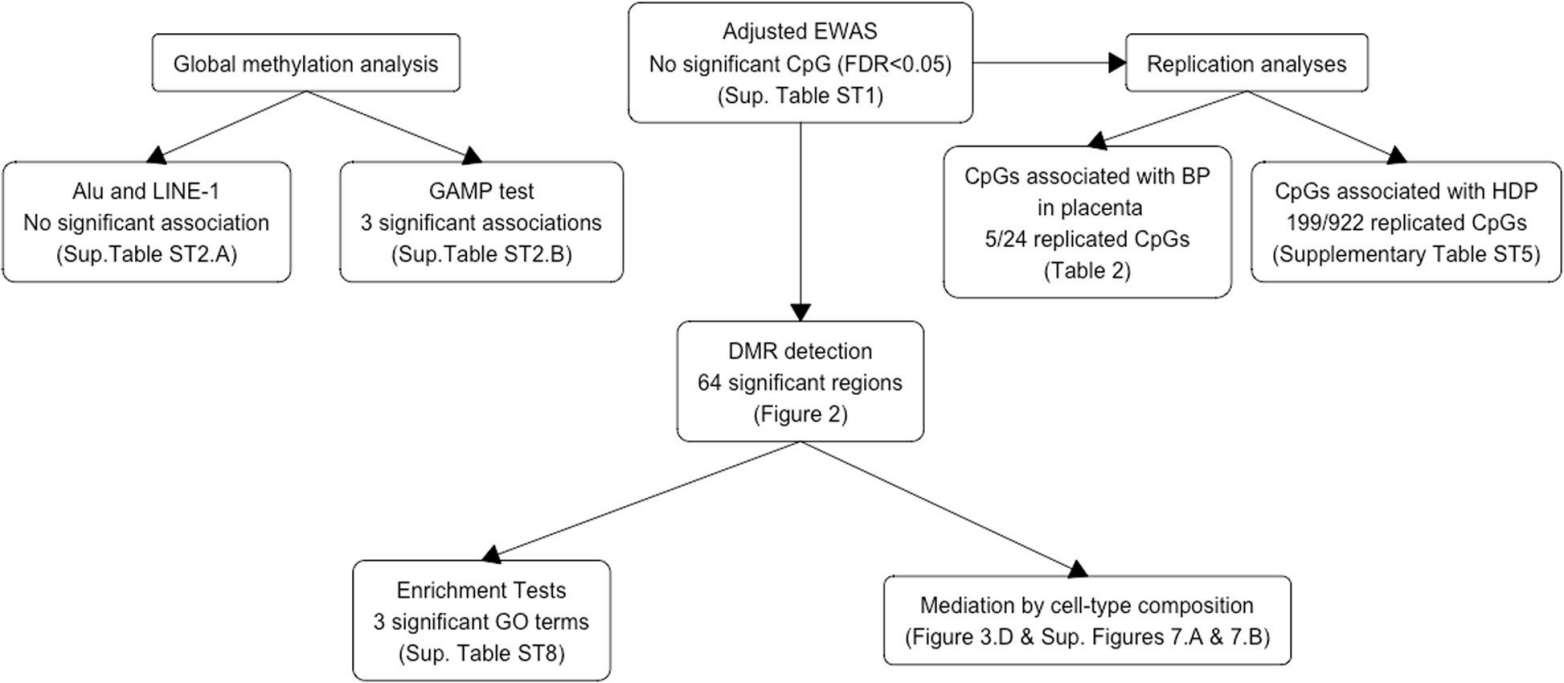
Workflow of the main statistical analyses performed in this study

The workflow of our main analyses is presented in Fig. 1. The association between maternal BP and DNAm was investigated at three levels: through global methylation analyses (Additional file 1: Methods) [31, 32], through epigenome-wide and regional analyses, and through an analysis of replication.

#### Summarizing BP during pregnancy

##### Indicators of blood pressure (BP)

In addition to the two usual measures of BP, diastolic and systolic blood pressure (DBP, SBP), we explored two indicators: mean arterial blood pressure (MAP) and pulse pressure (PP) defined respectively as: MAP = 1/3*SBP + 2/3*DBP and PP = SBP − DBP. MAP is an indicator of peripheral resistance and cardiac output [33]. PP is sometimes used as a surrogate for arterial stiffness, possibly reflecting maternal vascular adaptation to pregnancy and whose levels during early pregnancy were previously found associated with increased risk of preeclampsia [18, 19].

##### Maternal BP trajectory during pregnancy and considered time windows

Previous studies have highlighted peculiar non-constant and non-linear patterns of SBP and DBP variations during pregnancy [34, 35]. Hence, to examine the temporal behavior of the four BP indicators in the whole EDEN cohort (*n* = 1904), we relied on functional principal component analysis (fPCA) [36, 37] and the R package *fdapace* [38]. This method applies to sparse and irregular measurements and, among other things, provides a smooth estimate of the population average trajectory. Based on the fPCA results, we defined three different time windows over which average BP levels of each woman were computed: the *whole pregnancy*, *early pregnancy* (before the 27th week of gestation), and *late pregnancy* (from the 27th week of gestational to delivery) (Additional file 1: Figures S2.A-C).

##### Non-stationarity of BP and detrending

In the EDEN study, most BP measurements were abstracted from medical records and were thus irregularly collected among participants (Additional file 1: Figure S2.B). As BP levels undergo non-linear—even if slight—variations across pregnancy, using original measurements may introduce bias when computing each participant’s mean values over a given time window. To obtain comparable BP summaries, we computed and used detrended BP values for further statistical analyses (Additional file 1: Methods and Figures S2.A-D).

#### Association between BP and placental DNA methylation

Each association study between BP indicators and DNAm was performed for each specific time window of blood pressure change identified in fPCA (early, late, whole pregnancy) and adjusted for potential confounders and technical effects described below.

##### Adjustment for confounders and technical effects

Regressions of BP onto DNAm levels were adjusted for known potential confounders, selected a priori, including recruitment center (Nancy, Poitiers), maternal age (continuous), child sex, maternal education level (< 2 years after high school/ high school + 2 years/ ≥ high school + 3 years), maternal body mass index (BMI) before pregnancy (categorical: BMI < 18.5, 18.5 < BMI < 25, 25 < BMI < 30, BMI> 30) [39–43], parity (binary: 0, ≥ 1 child) [44, 45], and maternal active smoking (categorical: non-smoker, former smoker, smoked before and during pregnancy) [46]. Blood pressure shows strong seasonal fluctuations associated with environmental factors such as ambient temperature [47, 48], but there is little evidence for a clear or widespread association of meteorological factors or season of conception with placental DNAm [24]. As adjusting for a strong predictor of the exposure may induce a substantial bias, we chose not to adjust for such covariates which are tightly correlated with blood pressure but not or weakly associated with placental DNAm [49]. All models were further adjusted for known technical factors related to DNAm measurements (batch, plate, and chip).

##### Epigenome-wide association study

To evaluate the relationship between maternal BP indicators and placental DNAm of individual CpGs, while accounting for the repeated BP measurements, we assumed a two-stage mixed model for each CpG at a time [50]. Precisely, for each BP indicator, individual average detrended levels were first estimated using a mixed linear model with first-order time autocorrelation: BP = 1 + (1|id). In a second stage, these estimates (written $\hat{BP}$) were fitted in a robust linear regression model to test association with DNAm. Model and significance computations were made after converting methylation beta-values into *M*-values, owing to their better statistical properties [51]:

$${M}_{ij}={\mathit{\log}}_2\left(\frac{\beta_{ij}}{1-{\beta}_{ij}}\right)={\alpha}_{0j}+{\alpha}_{BPj}{\hat{BP}}_i+{\alpha}_{Zj}^T{Z}_i+{\varepsilon}_{ij}$$

where *β*_*ij*_ is the methylation beta-value for CpG *j* in subject *i*, *M*_*ij*_ is the corresponding *M*-value, *Z*_*i*_ is the set of adjustment factors, and *ε*_*ij*_ is the random error term. For the interpretability of associations, we used the *intercept* method to report effect sizes on beta values (Additional file 1: Methods) [52]. To account for multiple testing, we corrected *p*-values using the Benjamini-Hochberg method and considered a result significant when the false discovery rate (FDR) *p*-value was below 0.05. Moreover, we checked for genomic inflation and bias using the Bayesian method implemented in the R/Bioconductor package *BACON* [53].

##### Look-up into candidate lists in the placenta and cord blood

We sought whether our study in the EDEN cohort would replicate results from previous similar EWAS. To our knowledge, there is only one published EWAS, by Workalemahu et al., in which the association between continuous maternal SBP and DBP levels and DNAm from the fetal side of the placenta was explicitly examined, in a cohort of 301 participants [16]. This work highlighted 24 CpGs significantly associated with maternal BP levels. Other existing works related to maternal blood pressure during pregnancy are rather case-control studies focused either on hypertensive disorders during pregnancy (HDP) or on pre-eclampsia (PE). For comparison, we additionally performed a replication analysis of CpGs previously found associated with HDP (Additional file 1: Methods) [15, 54]. We considered as “significantly replicated” any candidate CpGs with matching direction of effect and whose uncorrected *p*-value was below 0.05.

##### Detection of differentially methylated regions (DMRs)

Based on recent guidelines, we made use of the *comb-p* method which was implemented in the R/Bioconductor package *ENmix* [55–57]. Using sliding windows, it aggregates CpG *p*-values computed in the EWAS accounting for probe spatial correlation across the genome [56]. Region *p*-values are then adjusted for multiple testing using the Benjamini-Hochberg method [57]. DMRs with FDR-adjusted *p*-value below 0.05 and including at least three probes at a maximum distance of 500 bp were considered significant. A maximal *p*-value of 0.001 was required to initiate a region. When possible, DMRs were then annotated to the nearest gene according to Illumina's annotation for the hg19 reference genome from the *IlluminaHumanMethylation450kanno.ilmn12.hg19* R/Bioconductor package, or to an overlapping gene from the more recent *ENSEMBL* annotation (Homo_sapiens.GRCh38.104.gtf). Otherwise, they were labeled as “intergenic” regions. Additionally, we searched for enriched specific genomic locations, associated traits, GO terms, and KEGG pathways among CpGs included in detected DMRs (Additional file 1: Methods) [58].

#### Sensitivity analyses

##### Gestational duration

Placental DNAm levels are known to be strongly associated with gestational duration. For this reason, we performed sensitivity analysis adjusting the robust regression models for gestational duration.

##### Preeclampsia

Women suffering from pre-eclampsia were shown to have characteristic BP patterns, especially a sharp rise during the 3rd trimester of pregnancy [34]. We performed a sensitivity analysis excluding the 17 preeclamptic mothers to investigate whether they drove the top hits or if some hits were also replicated in non-preeclamptic placentas.

#### Investigating the mediating role of cell-type composition

##### Role of cell-type composition

There is currently no consensus as to whether DNAm analyses should always be adjusted for cell mixtures [11, 12]. Holbrook et al. distinguished between two types of methylation signal: a *direct* or *canonical methylation signal*, that is independent of cellular heterogeneity, and an *indirect* or *extrinsic methylation signal*, that is due to differential mixtures of cell types. Both signals may be relevant and informative of the effect of maternal BP on placental DNAm. Indeed, hints at differences in placental morphology, development (like vessel formation) in women suffering from gestational hypertension, led us to hypothesize that cell type composition may be on the path between maternal blood pressure and placental DNAm [59, 60]. With this in mind, we sought to evaluate the role of placental cell-type components in the relation between BP indicators and DNAm levels. We therefore did not adjust for cell-type heterogeneity in the main analysis, but a sensitivity analyses adjusting for estimated cell mixture and mediation analyses were performed to investigate the direct and cell-composition-mediated effects of BP [61].

##### Estimating reference cell-type composition

Reference-based placental cell-type composition was estimated in silico using DNAm data, based on a recent work by Yuan et al. in which they measured the methylation profiles of six reference cell types (Endothelial, Hofbauer, nucleated Red Blood Cells (nRBC), Stromal, Syncytiotrophoblast, and Trophoblasts) in term placental tissues [20] (Additional file 1: Methods) [62–64].

##### Association between BP and placental cell-type composition

We first examined the possible influence of each maternal BP indicator on the *global* cellular composition through a multilinear regression model, adjusted for the confounders selected a priori. Association was tested for using an ANOVA [65]. In a second analysis, we examined whether some BP indicators were associated with specific reference cell types (Fig. 3 (C), Additional file 1: Methods and Figures S6.B) [66–68].

##### Mediation analyses

Postulating that maternal BP might have some effect on placental methylation through changes in cell composition, we performed mediation analyses in order to quantify the direct effect of BP on DNAm as well as its indirect effect mediated by cell-type composition. We considered two different approaches which both relied on two linear regression models: an exposure-mediator model and an outcome-mediator model [69, 70]. First, we made use of a novel method named *ccmm* (version 1.0, available on CRAN), which is able to handle multivariate compositional mediators as a whole and provides estimates for the direct and indirect effects along with component-wise indirect effects [71]*.* In a second analysis, we performed univariate mediation analyses to investigate the mediating role of the ratio of stromal cells over syncytiotrophoblasts. The ratio was first log-transformed and computations were carried out with the R package *mediation* [72]. Both mediation analyses were adjusted for the same confounders as those selected for the EWAS.

## Results

### Population characteristics

A total of 666 mother-child pairs were included in this study. They had similar distributions on considered covariates than the rest of the EDEN cohort (*n* = 1334, Additional file 2: Table ST1.A). On average, participating mothers were 29 years old, their gestational duration was 40 weeks, and 68.3% of them had normal BMI. Mean SBP and DBP over the whole pregnancy were 115 and 64.9 mmHg, respectively (Table 1). As expected in term placenta, syncytiotrophoblast was the predominant cell type (63% on average), the amounts of endothelial and stromal cells were similar (9% and 10%, respectively), and the contribution of Hofbauer cells and nRBC was very low (2%) (Additional file 2: Table ST1.B and Additional file 1: Figure S3) [20].

### Global methylation analyses

The average methylation level was 16.2 (±1.0) for *Alu* and 26.1 (± 1.9) for *LINE-1*. Analysis of *Alu* and *LINE-1* methylation levels yielded no significant differences related to any of the BP indicators considered therein (Additional file 2: Table ST2.A). However, in the GAMP analysis, the average levels of DBP and MAP during late pregnancy were significantly associated with the density of the DNAm distribution (*p*-value < 0.0033 and *p*-value < 0.0054, respectively) (Additional file 2: Table ST2.B).

### EWAS and replication analysis of individual CpGs

BIF coefficients were all close to 1 indicating no genomic inflation (Additional file 2: Table ST3); we therefore did not correct test *p*-values for genomic inflation. In the EWAS, no individual CpG was found significantly associated with any BP indicator after correcting for multiple tests (FDR < 0.05) (Additional file 2: Table ST4). Among the 24 CpG sites measured in the placenta previously found significantly associated with maternal SBP and/or DBP by Workalemahu et al. [16], 5 associations were replicated in our study with matching direction of association, and a sixth one was found significant but with diverging direction (Table 2). Two replicated CpGs, located on genes *NAV2* and *CAMK2B*, had been related to childhood acute lymphoblastic leukemia in a previous study [73]. Out of 922 CpG sites retrieved from the EWAS Atlas which were previously found significantly associated with hypertensive disorders in pregnancy, including preeclampsia, 228 CpGs were significantly associated with at least one BP indicator in our study (*p*-value < 0.05); these included 12 CpGs (out of 43) measured in cord blood and found significant in a broad meta-analysis by Kazmi et al. [15] (Additional file 2: Table ST5).

**Table 2:** Placental CpGs previously found significantly associated with maternal SBP and DBP during pregnancy which were replicated in our study

						Associated BP indicators in our study		
CpG	Position	Gene	Phenotype^**a**^	Trimester	Direction of Effects^**b**^	Early pregnancy	Late pregnancy	Pregnancy
cg05130406	chr12:75723912	*CAPS2*	SBP+DBP	3rd	1/1	-	SBP^c^; MAP^c^	SBP^c^
cg10752545	chr11:19734701	*NAV2*	SBP+DBP	3rd	1/1	-	DBP; MAP^c^	-
cg13534424	chr7:44365292	*CAMK2B*	SBP	1st and 3rd	1/1	SBP; DBP; MAP	SBP; DBP; MAP	SBP; DBP; MAP
cg23314283	chr13:53226751	*SUGT1*	SBP	1st	1/1	SBP; DBP; MAP	SBP; DBP; MAP	SBP; DBP; MAP
cg06880028	chr6:75918165	*Intergenic*	SBP+DBP	1st	1/1	-	-	PP; SBP^d^
cg02918224	chr20:33298052	*TP53INP2*	SBP+DBP	3rd; 2nd; and 3rd	-1/1	-	SBP; MAP^c^, PP^d^	SBP; MAP^c^

All 6 CpGs were replicated when further adjusting for cell-type composition and mediation analyses found no significant indirect effect of cell-type composition for theses CpGs

^a^Phenotypes CpG methylation was found associated with in the EWAS by Workalemahu et al. [16]

^b^Direction of effect observed in the study by Workalemahu et al. versus direction of effect observed in our study

^c^Significance was not reached anymore after excluding participants with preeclampsia

^d^Significance was reached only after excluding participants with preeclampsia

### Regional analysis

**Fig. 2:**
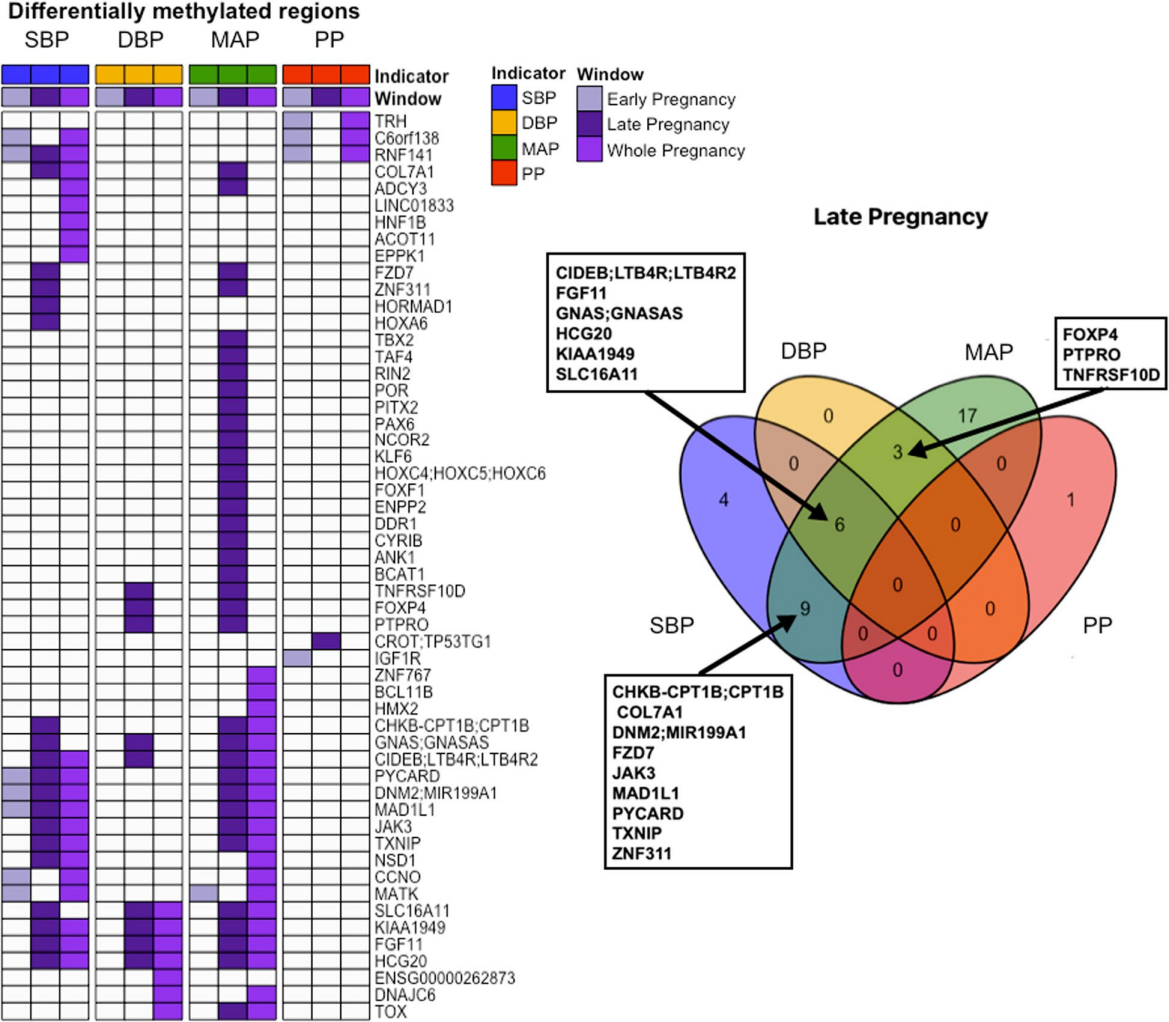
Differentially methylated regions in the placenta associated with maternal BP indicators average levels during pregnancy. DMRs were labeled according to their cognate genes. Only DMRs annotated to known genes are displayed. The Venn diagram on the right highlights DMRs found associated with at least two BP indicators during late pregnancy

Regional analyses identified 54 DMRs associated to known genes and 10 non-overlapping intergenic regions significantly associated (Sidak-corrected p-value below 0.05 and at least 3 probes) with the levels of at least one maternal BP indicator. Average MAP during late pregnancy and over the whole pregnancy produced the highest number of associations (35 and 20 annotated regions respectively), followed by SBP over the same time windows (19 and 20 annotated regions respectively) (Fig. 2 and Additional file 2: Table ST6). During late pregnancy, 3 DMRs were common to both MAP and DBP, 9 DMRs with both MAP and SBP, and 6 DMRs were associated with the three steady BP indicators (MAP, SBP, DBP) simultaneously (Fig. 2). Relatively few annotated DMRs were related to the average levels of SBP, DBP, and MAP during early pregnancy (7, 0, and 1, respectively). In contrast, PP yielded only 5 annotated DMRs and all except one were associated with early pregnancy levels.

**Fig. 3:**
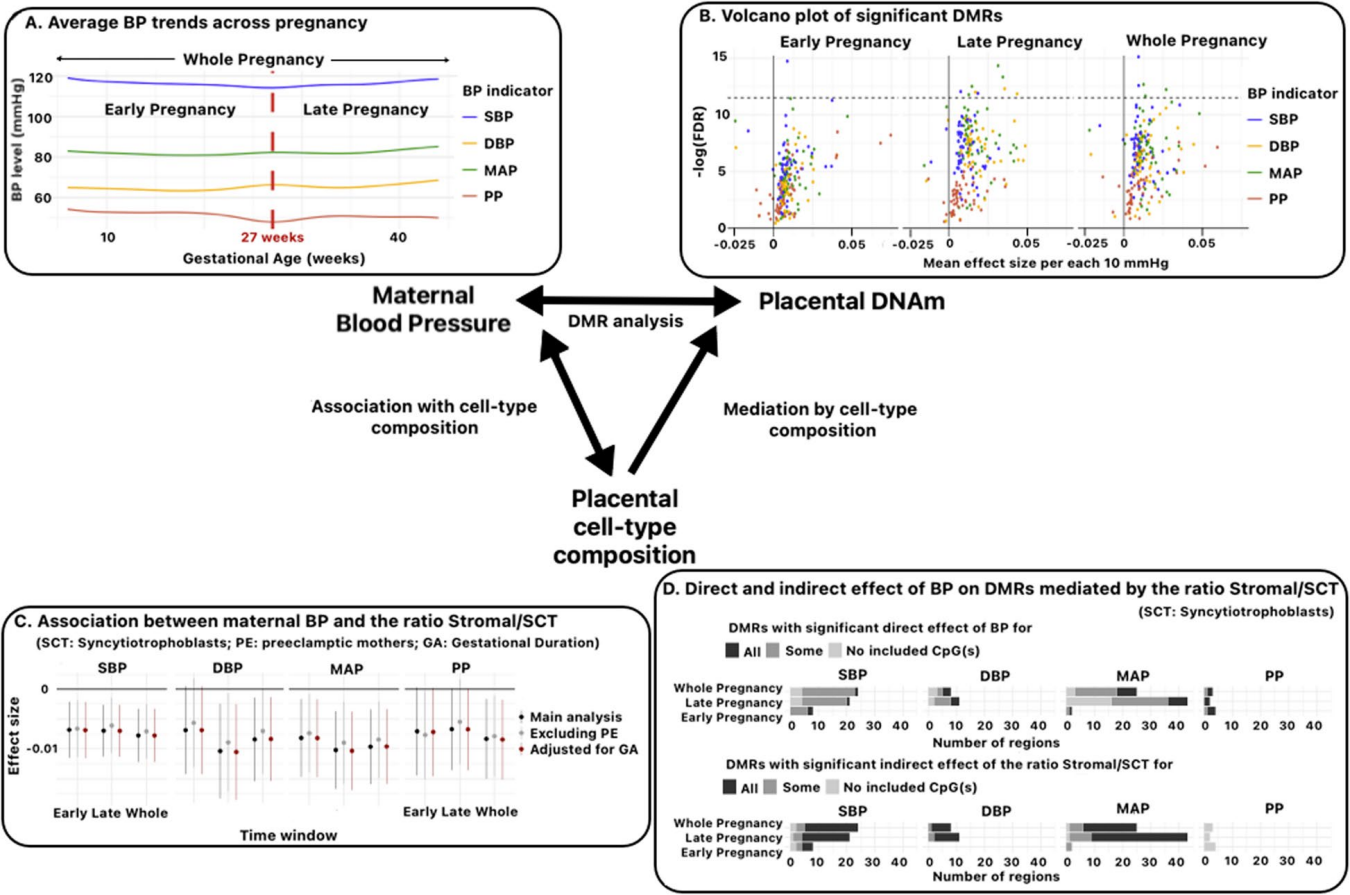
Graphical summary of the study. **A** Blood pressure (BP) trends across pregnancy. Four BP indicators were assessed from repeated SBP and DBP measurements during pregnancy and averaged within three time windows: early pregnancy (from conception to week 27), late pregnancy (from week 27 to delivery), and the whole pregnancy. **B** Volcano plots of differentially methylated regions (DMRs) significantly associated with maternal BP. The average effect sizes among CpGs included in detected DMRs are compared to the FDR *p*-value computed using the *comb-p* method. The dashed line indicates a threshold of 10E−6. **C** Effect sizes (and 95% CI) of the association between BP indicators and the ratio of stromal cells to syncytiotrophoblasts. Two additional sensitivity analyses were performed, one by excluding preeclamptic mothers (PE), the other one by further adjusting the models for gestational age (GA). **D** Mediation analysis of the role of the cell-type ratio stromal/syncytiotrophoblast in the association between maternal BP and detected DMRs. The direct and indirect effect (mediated by the cell-type ratio stromal/syncytiotrophoblast) of BP on DMRs were estimated CpG-wise and aggregated by DMR, thus defining three types of DMRs in both cases: those for which all CpGs have a significant effect (“All”), those for which no CpG has a significant effect (“No”), and those for which only a subset of CpGs have a significant effect (“Some”). Significance was determined from 95% bootstrap confidence intervals of the effect size

Average effect sizes among the 54 annotated DMRs ranged from −0.002 (corresponding to a decrease of 0.02 in the DNAm level of *MATK* for an increase of 10 mmHg in the average DBP or MAP during early pregnancy) to 0.007 (meaning an increase of 0.07 in the DNAm level of *TRH* for an increase of 10 mmHg in the average PP during early pregnancy). The average absolute effect size was 0.001 (Fig. 3 (B) and Additional file 2: Table ST6).

We note than even in the absence of detection, DMR average effect sizes were very similar among steady BP indicators but differed markedly from PP effect sizes (Additional file 1: Figure S4.B).

### Enrichment analyses of detected DMRs

For each BP indicator, we investigated whether genes annotated to the DMRs were enriched for GO terms and KEGG pathways (FDR < 0.05). We found enrichment in genes linked to molecular functions *leukotriene B4 receptor activity* and *leukotriene receptor activity* and to the biological process *leukotriene signaling pathway* for DBP, SBP, and MAP during late pregnancy, and also during pregnancy for SBP and MAP (Additional file 2: Table ST8.A). Additionally, DMRs detected for SBP, DBP, or MAP were significantly enriched in CpGs previously found associated with *metabolic traits*, nutrition (*high saturated fatty acids diet* and *fruit consumption*), respiratory traits (*lung function*, *lung cancer risk*, and *smoking*), and immune deficiencies and inflammation such as *HIV frailty*, *systemic lupus erythematosus*, *psoriasis, B acute lymphoblastic leukemia*, and various cancers (Additional file 2: Table ST7.B). Furthermore, during pregnancy and late pregnancy, CpGs included in DMRs were significantly more present around TSS for SBP and MAP and in gene body for DBP and MAP, suggesting a potential influence on the regulation of gene expression (Additional file 2: Table ST7.C).

### Sensitivity analyses

Overall, 35 out of 54 annotated DMRs were lost after exclusion of PE cases (Additional file 1: Figure S5.B and Additional file 2: Tables ST9.A1 and ST9.A2) and 8 annotated regions were no longer detected after adjustment for gestational age (Additional file 2: Tables ST9.B1 and ST9.B2), but, in both cases, the changes in average effect size were only slight (Additional file 1: Figure S5A) and the GO terms related to *leukotriene* were still found significantly enriched (Additional file 2: Tables ST8.B and ST8.C). In contrast, adjusting for reference cell-type composition led to substantial changes in average effect sizes and to a loss of significance of 44 out of 51 annotated DMRs associated with SBP, DBP, and MAP, but it did not affect the detection of regions associated with PP (Additional file 1: Figure S5A and Additional file 2: Tables ST9.C1 and ST9.C2). Adjusting for cell mixture highlighted only a few novel DMRs.

### Evaluating the role of cell-type composition

#### Association between BP and placental cell-type mixture

No clear and statistically significant influence of BP on the overall composition in cell types could be detected (Additional file 2: Table ST10.A). However, a cell-type by cell-type analysis revealed significant associations between SBP and MAP and the proportion of syncytiotrophoblast (SCT) and between all BP indicators’ levels and the estimated proportion of stromal tissue (Additional file 1: Figure 6.A and Additional file 2: Table ST10.B). Besides, a PCA performed on cell-type components pointed to a strong correlation between the proportions of stromal and SCT tissue (Additional file 1: Figure 6.B). Based on these considerations, we undertook further analysis of the link between the ratio Stromal/SCT and BP indicators, which indeed showed a significant or nearly significant association (*p*-value below 0.05) for all BP indicators over all time windows (Fig. 3 (C) and Additional file 2: Table ST10.C). In all cases, increase in average BP indicator levels was associated with lower values of the ratio Stromal/SCT.

#### Mediation analysis

Cell-type composition significantly (95% confidence intervals) mediated the effect of SBP, DBP, and MAP for at least one probe in more than half of the DMRs, with a stable magnitude of indirect effect sizes across CpGs (Additional file 1: Figure 7.A and Additional file 2: Table ST1). This proportion increased substantially when rather analyzing the mediating role of the ratio stromal/syncytiotrophoblast (Fig. 3 (D) and Additional file 2: Table ST12). Nonetheless, all three BP indicators still showed a significant direct effect on most evaluated probes and DMRs. Contrariwise, the relationship between PP and placental DNAm did not seem affected by cell-type heterogeneity and exhibited the strongest direct and total effect sizes estimated among all four BP indicators (Additional file 1: Figure 7.B). This suggests that, contrary to SBP, DBP, and MAP, PP may equally alter the methylation of all considered reference cell types in the detected DMRs.

## Discussion

We hypothesized that variations in maternal blood pressure during pregnancy could affect placental function and fetal development by altering placental DNAm. Accordingly, we investigated the association between four maternal blood pressure indicators over pregnancy and DNAm levels in human term placenta and examined the mediating role of cell-type composition, an indicator of cell differentiation and proper placental development. Our study is, to our knowledge, the first one to have explored and pointed out the discrepant influence of the pulsatile pressure indicator (PP) and steady BP indicators (SBP, DBP, and MAP) over placental DNAm and to have evaluated and highlighted the role played by cell-type composition in these relationships.

Only one EWAS of maternal SBP/DBP in the placenta has previously been reported and emphasized the association between maternal BP and the methylation of CpGs related to cardiometabolic traits [16]. Our study replicated one third of the CpGs found significant therein but there was no overlap with the 7 DMRs they detected. Moreover, we found a moderate overlap between our EWAS on individual CpGs in the placenta and those previously found associated with HDP in cord blood.

Our analyses did not detect strong and consistent global changes in placental DNAm associated with maternal BP and no significant CpG was found in the EWAS after correction for multiple testing (FDR < 0.05), but we found significant associations with regional changes. All in all, 64 DMRs were found associated to at least one BP indicator in our study. Steady blood pressure indicators SBP and MAP are tightly correlated. They thus yielded consistent results with a substantial overlap of the DMRs, mainly related to average BP levels observed during pregnancy and/or late pregnancy and moderate effect sizes. Interestingly, we observed more and stronger associations with MAP than with SBP or/and DBP. This could be explained by MAP being less dependent on measurement site or technique and least altered by measurement damping and would promote it as a valuable indicator for investigating the interplay between blood pressure and DNAm [74]. Furthermore, steady blood pressure indicators led to results globally distinct from those obtained with pulsatile blood pressure (PP). Associations between PP and DMRs were scarce but with stronger effect sizes. PP is defined as the difference between SBP and DBP; it therefore cumulates measurement noise from them both, which may partly explain the reduced number of significant results.

Pulse pressure was suggested to be a hallmark of maternal vascular adaptation to pregnancy and an early predictor of preeclampsia [18, 19]. Consistent with these, most significant results for PP in our study (4 out of 5 DMRs) were related to average levels during early pregnancy. Regions specific to PP included *TRH*, a gene implicated in the reduction of blood pressure in rats [75], and *IGF1R* a growth factor receptor which also participates in the regulation of blood pressure [76]. The two other DMRs were common with SBP: overlapping *RNF141* and *C6orf138* (*PTCHD4*), a gene found to be sensitive to transmitted adaptation to stress and associated with chronic obstructive pulmonary disease (COPD) [77, 78]. Accordingly, the strongest (total and direct) effect sizes of steady BP indicators on DMRs estimated through our mediation analyses pointed to genes pertaining to lung development and airway diseases: *CCNO* has been implicated in reduced generation of multiple motile cilia (*RGMC*) [79, 80], a chronic airway disease; *FOXF1* was involved in alveolar capillary dysplasia and pulmonary arterial hypertension [81]; *TBX2* is known to act on lung growth by maintaining cell proliferation in the embryonic lung mesenchyme [82, 83]; and *FOXP4* is a transcriptional repressor which regulates lung-specific gene expression. Consistent with these, DMR cognate genes were enriched in GO terms related to the leukotrienes, a family of molecules recognized to be mediators of pulmonary inflammation and thought to be critical in triggering acute asthma attacks and in causing longer-term hypersensitivity of the airways in chronic asthma [84]. These results are also in line with previous works showing that gestational hypertension is associated with increased production of leukotriene B4 in the placenta and suggesting that maternal blood pressure may influence lung function, wheezing, and asthma in children [8, 85]. In most cases, elevated maternal BP was correlated with increased DNAm levels, and DMRs overlapped TSS or/and gene bodies suggesting a link with the expression of related genes.

In EWAS, cell-type heterogeneity is often treated as a mere technical factor to be controlled for [12]. Yet, in some cases, it could also act as a mediator of an exposure over DNAm and be fruitfully investigated as such [11, 25]. Newly generated reference data allowed us to estimate cell-type proportions in silico and to formulate further biological interpretations.

The mediation analysis framework relied on the assumption that maternal blood pressure would have unilaterally influenced observed placental cell-type composition and DNAm. Though placental DNAm was measured from tissue extracted at birth, we cannot rule out reverse causation where placental state would have partly influenced maternal blood pressure. Nonetheless, we note that our major results still hold when we excluded preeclamptic participants from the EWAS. Moreover, these analyses allow us to estimate the proportion of the association between BP and DNAm which is due to variations in the cell-type composition. Our mediation analysis then indicates that maternal BP during pregnancy is doubly tied to term placental DNAm. On the one side, our results show that BP is associated with alterations in DNAm which may be partly explained by changes in placental cell-type composition, especially in the ratio of stromal to syncytiotrophoblast cells. This compares with recent observations showing that preeclampsia, a pregnancy disorder characterized by gestational hypertension, is associated with a lower amount and an altered structure and activity of mesenchymal stromal cells in term placental tissues [86, 87]. Interestingly, gestational age has also been associated with a decrease in the quantity of mesenchymal stromal cells and an increase in syncytiotrophoblasts, suggesting that elevated maternal blood pressure could be associated with an acceleration of placental cellular maturation [14, 20]. On the other side, we noticed that, even after accounting for cell-type mediation, all BP indicators kept significant direct association with DNAm (thus independent of cell-type distribution). As to the mediating role of cell mixtures, steady and pulsatile BP indicators also showed distinct behaviors. We found a significant indirect effect of cell-type composition in our analyses of SBP, DBP, and MAP, which may indicate that steady blood pressure differentially influences the methylation of the various reference cell types. In contrast, cell-type composition showed no significant indirect effect in the relationship between pulse pressure and DNAm, which we interpreted as PP equally influencing the methylation of all considered reference cell types.

We acknowledge several strengths and limitations to the present study. First, variations in blood pressure measurements, due to irregular consultations across participants, technical and circadian variability, likely impaired our capacity to discover true associations [88, 89]. Nonetheless, we tried to mitigate potential biases by relying on detrended BP measurements that we averaged over critical stages of pregnancy. Second, though we were able to explicitly control for a large set of acknowledged confounders such as maternal pre-gravid BMI and tobacco consumption, possible residual confounding due to factors which are difficult to assess, such as maternal alcohol consumption or diet during pregnancy, may remain [43]. However, our models were adjusted for maternal education level, a proxy for mothers’ socio-economic level which is correlated with these behaviors. Third, cell-type components were only in silico estimates derived from DNAm data. Yuan et al*.* acknowledged that reference cell-type definition is arguable and limited by experimental capacity to identify and isolate more subtypes. Cell types with clearly distinct histological profiles, such as trophoblasts and syncytiotrophoblasts, may also be difficult to discriminate from their methylation patterns, which could bias the estimation of their respective proportions [20]. That said, reference-based estimates greatly improve over reference-free data in that they provide cell-mixture estimates independent of other sources of confusion and whose components can be biologically interpreted [14]. These assets were crucial for our analysis of mediation and our ability to specifically point out which cell types were more likely involved in the association between maternal BP and placental methylation. Finally, for DNAm is thought to be involved in the regulation of gene expression, our approach had a focus on DMRs located nearby known genes. Though the differences detected in methylation levels were small and, while significant, may not greatly contribute to differential expression of genes, this method may still prove to be useful for discovering epigenetic markers of placental stress related to maternal blood pressure. One must note that our work focused on one specific type of epigenetic markers. Future studies investigating and integrating other epigenetic mechanisms, such as histone modifications and microRNA, will be helpful to decipher the complexity of the interplay between maternal blood pressure and placental epigenetic changes [10].

## Conclusions

We studied and compared the association of the four most common maternal hemodynamic parameters during pregnancy with placental DNA methylation at birth and pointed out that, in addition to SBP and DBP, maternal MAP and PP were worth being investigated and showed markedly distinct behaviors. Our study confirmed that steady blood pressure during pregnancy is associated with alterations in placental methylation and further highlights that this relationship is twofold: both direct, potentially affecting all cell types, and indirect through changes in cell-type composition. More specifically, an increase in maternal blood pressure was associated with a decrease in the ratio between stromal cells and syncytiotrophoblasts, even in absence of preeclampsia, suggesting that maternal blood pressure could be a primary determinant of alterations in the maturation of the placenta. Moreover, elevated blood pressure was associated with increased methylation of genomic regions involved in airway development and disease. These results could provide clues to enlighten the correlations hitherto noticed between maternal hypertension and airway diseases in children and inform recommendations for reducing maternal stress during pregnancy.

## Supplementary Information

**Additional file 1: Supplementary methods and figures S1-S7.** S1 - Workflow of DNA methylation data pre-processing; S2 - Overview of blood pressure data; S3 - Reference placental cell-type composition observed in the EDEN cohort; S4 - Pearson correlation between DMR effect sizes; S5.A - Sensitivity analyses for DMRs found in our main analysis; S5.B - Sensitivity analysis excluding preeclampsia cases for the DMRs found in our main analysis; S6.A - Magnitude and 95% confidence intervals of the association between each BP indicator and each reference cell-type; Figure S6.B - Compositional Principal Component Analysis performed on cell-type mixtures; S7.A - Diagnostic of the direct and indirect effect (mediated by cell-type composition) of BP on the methylation level of DMRs; S7.B - Total, direct and indirect effect of BP on the methylation level of DMRs.

**Additional file 2: Supplementary tables ST1-ST12.** ST1 – Participants’ characteristics; ST2 - Global methylation analyses; ST3 - Inflation Factors; ST4 - Top 100 DMPs; ST5 - Replication Analysis; ST6 - DMRs; ST7 - Trait Enrichment; ST8 - Gene enrichment; ST9 - Sensitivity analyses of DMRs; ST10 - Association between blood pressure and cell-type composition; ST11 – Compositional mediation analysis; ST12 - Mediation analysis: role of the ratio Stromal/Syncytiotrophoblasts.

## Data Availability

The EDEN datasets analyzed in the presented study are not publicly available as they are containing information that could compromise the research participant’s privacy/consent. However, they are available on reasonable request and with permission from the EDEN Steering Committee by filling the questionnaire at http://eden.vjf.inserm.fr/en/page/25/submit-a-research-project. The code used to generate the results is available at https://github.com/lbroseus.

## Abbreviations

BP: Blood pressure
CI: Confidence interval
DBP: Diastolic blood pressure
DNAm: DNA methylation
EWAS: Epigenome-wide Association Study
FDR: False discovery rate
HDP: Hypertensive disorders of pregnancy
MAP: Mean arterial pressure
PE: Preeclampsia
PP: Pulse pressure
SBP: Systolic blood pressure
SCT: Syncytiotrophoblasts

## References

[1] Murphy VE, Smith R, Giles WB, Clifton VL (2006). Endocrine regulation of human fetal growth: the role of the mother, placenta, and fetus. Endocr Rev.

[2] Huppertz B (2008). Placental origins of preeclampsia. Hypertension..

[3] Roberts JM, Gammill HS (2005). Preeclampsia. Hypertension.

[4] Atlass J, Menke M, Parks WT, Catov JM (2020). Pre-conception blood pressure and evidence of placental malperfusion. BMC Pregnancy Childbirth.

[5] Seely EW, Ecker J (2014). Chronic Hypertension in Pregnancy. Circulation..

[6] Bramham K, Parnell B, Nelson-Piercy C, Seed PT, Poston L, Chappell LC (2014). Chronic hypertension and pregnancy outcomes: systematic review and meta-analysis. BMJ..

[7] Bakker R, Steegers EAP, Hofman A, Jaddoe VWV (2011). Blood pressure in different gestational trimesters, fetal growth, and the risk of adverse birth outcomes: the generation R study. Am J Epidemiol.

[8] Wilmink FA, den DHT, de JJC, Reiss IKM, Jaddoe VWV, Steegers EA, et al. Maternal blood pressure and hypertensive disorders during pregnancy and childhood respiratory morbidity: the Generation R Study. Eur Respir J. 2018;52(5). 10.1183/13993003.00378-2018 30309974

[9] Vlahos A, Mansell T, Saffery R, Novakovic B (2019). Human placental methylome in the interplay of adverse placental health, environmental exposure, and pregnancy outcome. PLoS Genet.

[10] Apicella C, Ruano CSM, Méhats C, Miralles F, Vaiman D (2019). The role of epigenetics in placental development and the etiology of preeclampsia. Int J Mol Sci.

[11] Holbrook JD, Huang RC, Barton SJ, Saffery R, Lillycrop KA (2017). Is cellular heterogeneity merely a confounder to be removed from epigenome-wide association studies?. Epigenomics..

[12] Jaffe AE, Irizarry RA (2014). Accounting for cellular heterogeneity is critical in epigenome-wide association studies. Genome Biol.

[13] Campbell KA, Colacino JA, Park SK, Bakulski KM (2020). Cell types in environmental epigenetic studies: biological and epidemiological frameworks. Curr Envir Health Rpt.

[14] Dieckmann L, Cruceanu C, Lahti-Pulkkinen M, Lahti J, Kvist T, Laivuori H (2022). Reliability of a novel approach for reference-based cell type estimation in human placental DNA methylation studies. Cell Mol Life Sci.

[15] Kazmi N, Sharp GC, Reese SE, Vehmeijer FO, Lahti J, Page CM (2019). Hypertensive disorders of pregnancy and DNA methylation in newborns. Hypertension..

[16] Tsegaselassie W, Marion O, Shrestha Deepika W, Jing GK, L., Tekola-Ayele Fasil. (2020). Differential DNA methylation in placenta associated with maternal blood pressure during pregnancy. Hypertension..

[17] Darne B, Girerd X, Safar M, Cambien F, Guize L (1989). Pulsatile versus steady component of blood pressure: a cross-sectional analysis and a prospective analysis on cardiovascular mortality. Hypertension..

[18] Thadhani R, Ecker JL, Kettyle E, Sandler L, Frigoletto FDJ (2001). Pulse pressure and risk of preeclampsia: a prospective study. Obstet Gynecol.

[19] Elvan-Taşpinar A, Franx A, Bots ML, Koomans HA, Bruinse HW (2005). Arterial stiffness and fetal growth in normotensive pregnancy. Am J Hypertens.

[20] Yuan V, Hui D, Yin Y, Peñaherrera MS, Beristain AG, Robinson WP (2021). Cell-specific characterization of the placental methylome. BMC Genomics.

[21] Heude B (2016). Cohort Profile: The EDEN mother-child cohort on the prenatal and early postnatal determinants of child health and development. Int J Epidemiol.

[22] Hampel R, Lepeule J, Schneider A, Bottagisi S, Charles MA, Ducimetière P (2011). Short-term impact of ambient air pollution and air temperature on blood pressure among pregnant women. Epidemiology..

[23] Bronsert MR, Henderson WG, Valuck R, Hosokawa P, Hammermeister K (2013). Comparative effectiveness of antihypertensive therapeutic classes and treatment strategies in the nitiation of therapy in primary care patients: a Distributed Ambulatory Research in Therapeutics Network (DARTNet) Study. J Am Board Fam Med.

[24] Abraham E, Rousseaux S, Agier L, Giorgis-Allemand L, Tost J, Galineau J (2018). Pregnancy exposure to atmospheric pollution and meteorological conditions and placental DNA methylation. Environ Int.

[25] Jedynak P, Tost J, Calafat AM, Bourova-Flin E, Busato F, Forhan A (2021). Pregnancy exposure to synthetic phenols and placental DNA methylation — An epigenome-wide association study in male infants from the EDEN cohort. Environ Pollut.

[26] Teschendorff AE, Marabita F, Lechner M, Bartlett T, Tegner J, Gomez-Cabrero D (2013). A beta-mixture quantile normalization method for correcting probe design bias in Illumina Infinium 450 k DNA methylation data. Bioinformatics..

[27] Peters TJ, Buckley MJ, Statham AL, Pidsley R, Samaras K, V Lord R (2015). De novo identification of differentially methylated regions in the human genome. Epigenetics Chromatin.

[28] Hop PJ, Zwamborn RAJ, Hannon EJ, Dekker AM, van Eijk KR, Walker EM, et al. Cross-reactive probes on Illumina DNA methylation arrays: a large study on ALS shows that a cautionary approach is warranted in interpreting epigenome-wide association studies. NAR Genomics Bioinformatics. 2020;2(lqaa105). 10.1093/nargab/lqaa105 PMC7745769 33554115

[29] Benton MC, Johnstone A, Eccles D, Harmon B, Hayes MT, Lea RA (2015). An analysis of DNA methylation in human adipose tissue reveals differential modification of obesity genes before and after gastric bypass and weight loss. Genome Biol.

[30] Chen Y a, Lemire M, Choufani S, Butcher DT, Grafodatskaya D, Zanke BW (2013). Discovery of cross-reactive probes and polymorphic CpGs in the Illumina Infinium HumanMethylation450 microarray. Epigenetics..

[31] Yang AS, Estécio MRH, Doshi K, Kondo Y, Tajara EH, Issa JJ (2004). A simple method for estimating global DNA methylation using bisulfite PCR of repetitive DNA elements. Nucleic Acids Res.

[32] Zhao N, Bell DA, Maity A, Staicu AM, Joubert BR, London SJ (2015). Global analysis of methylation profiles from high resolution CpG data. Genet Epidemiol.

[33] Franklin SS, Lopez VA, Wong ND, Mitchell GF, Larson MG, Vasan RS (2009). Single versus combined blood pressure components and risk for cardiovascular disease. Circulation..

[34] Corrie M-W, Lawlor DA, Abigail F, Margaret M, Nelson SM, Kate T (2012). Blood pressure change in normotensive, gestational hypertensive, preeclamptic, and essential hypertensive pregnancies. Hypertension..

[35] Loerup L, Pullon RM, Birks J, Fleming S, Mackillop LH, Gerry S (2019). Trends of blood pressure and heart rate in normal pregnancies: a systematic review and meta-analysis. BMC Med.

[36] Chen K, Zhang X, Petersen A, Müller HG (2017). Quantifying infinite-dimensional data: functional data analysis in action. Stat Biosci.

[37] Shen M, Tan H, Zhou S, Smith GN, Walker MC, Wen SW (2017). Trajectory of blood pressure change during pregnancy and the role of pre-gravid blood pressure: a functional data analysis approach. Sci Rep.

[38] Gajardo A, Carroll C, Chen Y, Dai X, Fan J, Hadjipantelis PZ (2021). fdapace: functional data analysis and empirical dynamics.

[39] Gaillard R, Steegers EA, Hofman A, Jaddoe VW (2011). Associations of maternal obesity with blood pressure and the risks of gestational hypertensive disorders. The Generation R Study. J Hypertens.

[40] Miller RS, Thompson ML, Williams MA (2007). Trimester-specific blood pressure levels in relation to maternal pre-pregnancy body mass index. Paediatr Perinat Epidemiol.

[41] Nogues P, Dos Santos E, Jammes H, Berveiller P, Arnould L, Vialard F (2019). Maternal obesity influences expression and DNA methylation of the adiponectin and leptin systems in human third-trimester placenta. Clin Epigenetics.

[42] Shrestha D, Ouidir M, Workalemahu T, Zeng X, Tekola-Ayele F (2020). Placental DNA methylation changes associated with maternal prepregnancy BMI and gestational weight gain. Int J Obes.

[43] Thakali KM, Zhong Y, Cleves M, Andres A, Shankar K (2020). Associations between maternal body mass index and diet composition with placental DNA methylation at term. Placenta..

[44] Macdonald-Wallis C, Tilling K, Fraser A, Nelson SM, Lawlor DA (2011). Established preeclampsia risk factors are related to patterns of blood pressure change in normal term pregnancy: findings from the Avon Longitudinal Study of Parents and Children. J Hypertens.

[45] Simpkin AJ, Durban M, Lawlor DA, MacDonald-Wallis C, May MT, Metcalfe C (2018). Derivative estimation for longitudinal data analysis: examining features of blood pressure measured repeatedly during pregnancy. Stat Med.

[46] Bakker R, Steegers EA, Mackenbach JP, Hofman A, Jaddoe VW (2010). Maternal smoking and blood pressure in different trimesters of pregnancy: The Generation R Study. J Hypertens.

[47] Brennan PJ, Greenberg G, Miall WE, Thompson SG (1982). Seasonal variation in arterial blood pressure. Br Med J (Clin Res Ed).

[48] Madsen C, Nafstad P (2006). Associations between environmental exposure and blood pressure among participants in the Oslo Health Study (HUBRO). Eur J Epidemiol.

[49] Pearl J. On a class of bias-amplifying variables that endanger effect estimates. arXiv. 2012:12033503.

[50] Sayers A, Heron J, Smith A, Macdonald-Wallis C, Gilthorpe M, Steele F (2017). Joint modelling compared with two stage methods for analysing longitudinal data and prospective outcomes: A simulation study of childhood growth and BP. Stat Methods Med Res.

[51] Du P, Zhang X, Huang CC, Jafari N, Kibbe WA, Hou L (2010). Comparison of Beta-value and M-value methods for quantifying methylation levels by microarray analysis. BMC Bioinformatics.

[52] Kruppa J, Sieg M, Richter G, Pohrt A (2021). Estimands in epigenome-wide association studies. Clin Epigenetics.

[53] van Iterson M, van Zwet EW, Heijmans BT, the BIOS Consortium (2017). Controlling bias and inflation in epigenome- and transcriptome-wide association studies using the empirical null distribution. Genome Biol.

[54] Xiong Z, Li M, Yang F, Ma Y, Sang J, Li R (2020). EWAS Data Hub: a resource of DNA methylation array data and metadata. Nucleic Acids Res.

[55] Mallik S, Odom GJ, Gao Z, Gomez L, Chen X, Wang L (2019). An evaluation of supervised methods for identifying differentially methylated regions in Illumina methylation arrays. Brief Bioinform.

[56] Pedersen BS, Schwartz DA, Yang IV, Kechris KJ (2012). Comb-p: software for combining, analyzing, grouping and correcting spatially correlated P-values. Bioinformatics..

[57] Xu Z, Xie C, Taylor JA, Niu L (2021). ipDMR: identification of differentially methylated regions with interval P-values. Bioinformatics..

[58] Phipson B, Maksimovic J, Oshlack A (2016). missMethyl: an R package for analyzing data from Illumina’s HumanMethylation450 platform. Bioinformatics..

[59] Chhatwal J, Chaudhary DN, Chauhan N (2018). Placental changes in hypertensive pregnancy: a comparison with normotensive pregnancy. Int J Reprod Contracept Obstet Gynecol.

[60] Krielessi V, Papantoniou N, Papageorgiou I, Chatzipapas I, Manios E, Zakopoulos N (2012). Placental pathology and blood pressure’s level in women with hypertensive disorders in pregnancy. Obstet Gynecol Int.

[61] VanderWeele TJ, Mumford SL, Schisterman EF (2012). Conditioning on intermediates in perinatal epidemiology. Epidemiology..

[62] Teschendorff AE, Breeze CE, Zheng SC, Beck S (2017). A comparison of reference-based algorithms for correcting cell-type heterogeneity in Epigenome-Wide Association Studies. BMC Bioinformatics.

[63] Hron K, Templ M, Filzmoser P (2010). Imputation of missing values for compositional data using classical and robust methods. Comput Stat Data Analysis.

[64] Templ M, Hron K, Filzmoser P (2011). robCompositions: an R-package for robust statistical analysis of compositional data. Compositional Data Analysis.

[65] van den Boogaart KG, Tolosana-Delgado R, van den Boogaart KG, Tolosana-Delgado R (2013). Linear models for compositions. Analyzing Compositional Data with R [Internet].

[66] Barton SJ, Melton PE, Titcombe P, Murray R, Rauschert S, Lillycrop KA (2019). In epigenomic studies, including cell-type adjustments in regression models can introduce multicollinearity, resulting in apparent reversal of direction of association. Front Genet.

[67] Hron K, Filzmoser P, Thompson K (2012). Linear regression with compositional explanatory variables. J Appl Stat.

[68] van den Boogaart KG, Filzmoser P, Hron K, Templ M, Tolosana-Delgado R (2021). Classical and robust regression analysis with compositional data. Math Geosci.

[69] Baron RM, Kenny DA (1986). The moderator–mediator variable distinction in social psychological research: conceptual, strategic, and statistical considerations. J Pers Soc Psychol.

[70] MacKinnon DP (2008). Introduction to statistical mediation analysis.

[71] Sohn MB, Li H (2019). Compositional mediation analysis for microbiome studies. Ann Appl Stat.

[72] Tingley D, Yamamoto T, Hirose K, Keele L, Imai K (2014). mediation: R Package for Causal Mediation Analysis. J Stat Softw.

[73] Gabriel AS, Lafta FM, Schwalbe EC, Nakjang S, Cockell SJ, Iliasova A (2015). Epigenetic landscape correlates with genetic subtype but does not predict outcome in childhood acute lymphoblastic leukemia. Epigenetics..

[74] Sturgess DJ, Bersten AD, Soni N (2014). 16 - Haemodynamic monitoring. Oh’s Intensive Care Manual (Seventh Edition).

[75] García SI, Alvarez AL, Porto PI, Garfunkel VM, Finkielman S, Pirola CJ (2001). Antisense inhibition of thyrotropin-releasing hormone reduces arterial blood pressure in spontaneously hypertensive rats. Hypertension..

[76] Cirrik S, Schmid-Schönbein GW (2018). IGF-1 receptor cleavage in hypertension. Hypertens Res.

[77] Burton NO, Willis A, Fisher K, Braukmann F, Price J, Stevens L, et al. Intergenerational adaptations to stress are evolutionarily conserved, stress-specific, and have deleterious trade-offs. Tautz D, Wittkopp PJ, editors. eLife. 2021;10:e73425. 10.7554/eLife.73425 PMC8570697 34622777

[78] Morrow JD, Cho MH, Hersh CP, Pinto-Plata V, Celli B, Marchetti N (2016). DNA methylation profiling in human lung tissue identifies genes associated with COPD. Epigenetics..

[79] Wallmeier J, Al-Mutairi DA, Chen CT, Loges NT, Pennekamp P, Menchen T (2014). Mutations in CCNO result in congenital mucociliary clearance disorder with reduced generation of multiple motile cilia. Nat Genet.

[80] Amirav I, Wallmeier J, Loges NT, Menchen T, Pennekamp P, Mussaffi H (2016). Systematic analysis of CCNO variants in a defined population: implications for clinical phenotype and differential diagnosis. Hum Mutat.

[81] Sen P, Yang Y, Navarro C, Silva I, Szafranski P, Kolodziejska KE (2013). Novel FOXF1 mutations in sporadic and familial cases of alveolar capillary dysplasia with misaligned pulmonary veins imply a role for its DNA binding domain. Hum Mutat.

[82] Lüdtke TH, Rudat C, Wojahn I, Weiss AC, Kleppa MJ, Kurz J (2016). Tbx2 and Tbx3 act downstream of Shh to maintain canonical Wnt signaling during branching morphogenesis of the murine lung. Dev Cell.

[83] Wojahn I, Lüdtke TH, Christoffels VM, Trowe MO, Kispert A (2019). TBX2-positive cells represent a multi-potent mesenchymal progenitor pool in the developing lung. Respir Res.

[84] Berger A (1999). What are leukotrienes and how do they work in asthma?. BMJ.

[85] Biagi G, Rosa VD, Pelusi G, Scagliarini G, Sani G, Coccheri S (1990). Increased placental production of leukotriene B4 in gestational hypertension. Thromb Res.

[86] Campbell KA, Colacino JA, Puttabyatappa M, Dou JF, Elkin ER, Hammoud SS, et al. Placental gene expression-based cell type deconvolution: cell proportions drive preeclampsia gene expression differences. bioRxiv. 2021; 2021.07.29.454041. 10.1038/s42003-023-04623-6 PMC10011423 36914823

[87] Romberg SI, Kreis NN, Friemel A, Roth S, Souto AS, Hoock SC (2022). Human placental mesenchymal stromal cells are ciliated and their ciliation is compromised in preeclampsia. BMC Med.

[88] Roberts ML, Kotchen TA, Pan X, Li Y, Yang C, Liu P (2022). Unique associations of DNA methylation regions with 24-hour blood pressure phenotypes in blacks. Hypertension.

[89] Vaiman D (2022). White-coat free genome-wide epigenetics of human blood pressure. Hypertension.

