# Supplementary material for: Maternal blood pressure associates with placental DNA methylation both directly and through alterations in cell-type composition
Source: BMC Med. 2022 Oct 20;20:397. doi: 10.1186/s12916-022-02610-y (PMC9585724; doi:10.1186/s12916-022-02610-y)
Supplement: Supplementary file 1 — Additional file 1: Supplementary methods and figures S1-S7. S1 - Workflow of DNA methylation data pre-processing; S2 - Overview of blood pressure data; S3 - Reference placental cell-type composition observed in the EDEN cohort; S4 - Pearson correlation between DMR effect sizes; S5.A - Sensitivity analyses for DMRs found in our main analysis; S5.B - Sensitivity analysis excluding preeclampsia cases for the DMRs found in our main analysis; S6.A - Magnitude and 95% confidence intervals of the association between each BP indicator and each reference cell-type; Figure S6.B - Compositional Principal Component Analysis performed on cell-type mixtures; S7.A - Diagnostic of the direct and indirect effect (mediated by cell-type composition) of BP on the methylation level of DMRs; S7.B - Total, direct and indirect effect of BP on the methylation level of DMRs. [file 12916_2022_2610_MOESM1_ESM.docx]

**Supplementary Material**

**Methods**

**Supplementary Figure 1. Workflow of DNA methylation data pre-processing.** DNAm data were generated using the Illumina 450K array. All samples passed initial quality control and had on average > 98% of valid data points (detection p-value < 0.01, N = 668). One sample was then excluded due to detected sex mix-up (N = 667). Methylation beta values were normalized with the BMIQ method.

**
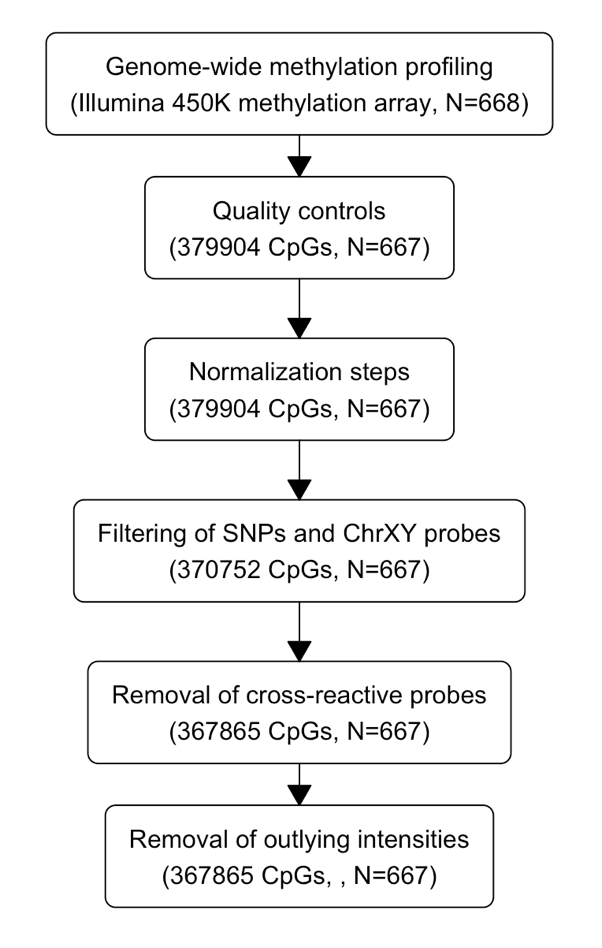
**

**Global methylation analysis**

We interrogated the impact of BP on the overall methylation level using two different approaches: one relying on repetitive DNA elements *Alu* and *LINE-1* as surrogate markers of genome-wide methylation change (1), the second one based on the global distribution of methylation levels estimated for the 450K Illumina array. Association of *Alu*, *LINE-1* and with each of the four BP indicators was examined using robust linear regressions, adjusted for the same confounders selected for the EWAS and relevant technical effects (batch and plate). The global analysis of methylation profiles was implemented using the *GAMP* method and its accompanying R package (2). In this approach, each participant’s methylation profile is represented by the empirical density function (or the cumulative distribution function) of the individual CpG methylation levels, which is in turn summarized with B-spline coefficients. A variance component score test is then computed to test the association between B-spline coefficients and each BP indicator separately, while adjusting for confounders and technical effects (batch, chip and plate).

**Non-stationarity of BP and detrending**

To obtain comparable BP summaries, we sought to estimate and cancel out the non-linear time trend. For so doing, each individual BP value was centered according to the value expected at the same timepoint. In the absence of BP standards during pregnancy, we made use of the extended EDEN cohort (n=1904) itself to compute these reference BP values by computing robust moving average (excluding the 5% most extreme values on both sides) over a window of one month (five weeks) around the gestational week of each BP measurement. Only normotensive mothers without a history of hypertension were included to estimate monthly means **(Supplementary Figure 2.A-C)**. Further statistical analyses were performed using detrended BP values **(Supplementary Figure 2.D)**.

**Supplementary Figure 2. Overview of blood pressure (BP) data** in the extended EDEN cohort (1904 individuals with at least one SBP and one DBP measurement). **A.** Distributions of original BP values. **B.** Number of participants with at least one SBP/DBP measurement and the resulting number of SBP/DBP measurements available per month of pregnancy. **C**. Average population BP trajectories estimated using functional PCA (*fPCA*). **D.** Detrended BP values, obtained by subtracting the population monthly mean from original values.

**Back-transformation to beta-values**

To report the effect sizes on beta values, the regression coefficients estimated on M-values were transformed back using the formula given by the *intercept method* which accounts for confounders (3):

$\beta_{BP} = g(\hat{\alpha_{0}} + \hat{\alpha_{BP}}) - g(\hat{\alpha_{0}}$)

Where $g(m) = \frac{2^{m}}{2^{m}+1}$ is the inverse transformation from M-values to beta-values and the hat symbol stands for estimates computed from the previous linear regression model.

**Look-up into candidate lists**

We queried the EWAS Atlas database for CpGs previously found associated with the trait “maternal hypertensive disorders during pregnancy”, including preeclampsia, which amounted to 922 probes which were measured either in placenta samples or cord blood (4). These included 42 significant probes from a broad meta-analysis in *cord blood* by *Kazmi* et al. (5).

**Enrichment analyses**

The EWAS Atlas records results from previously published EWAS allowing to link each CpGs to a list of traits they were found associated with. Relying on this database and the EWAS toolkit (https://ngdc.cncb.ac.cn/ewas/toolkit), we queried, for each BP indicator and each time window, whether the set of CpGs included in a DMR were significantly enriched in specific traits. The same tool was used to test for enrichment in specific genomic locations such as Transcription Start Sites (TSS), UTR or CpG Islands. At least 20 probes were necessary to perform the enrichment test. Additionally, we searched for enriched GO terms and KEGG pathways among CpGs included in detected DMRs using the dedicated function *goregion()* from the R package *missMethyl* which enables to test for gene set enrichment while correcting for the unbalanced number of probes per gene on the 450K array (6).

**Estimating reference cell-type composition**

Taking advantage of the cell-type specific reference provided in the R package *planet*, we applied the Robust Partial Correlations (RPC) method implemented in the R package *EpiDISH* (57) to our methylation data. If a value of zero was obtained for a cell type, it was considered as below the limit of detection and a non-zero value was imputed using the *impCoda* function from the R package *robCompositions* designed for compositional data and which relies on an iterative regression-based procedure after KNN-initialization (58,59).

**Adjusting for cell-type composition in linear regression models.** A cell composition can be represented as a vector of mutually dependent proportions summing up to one. To avoid multicollinearity and instability of the results, each vector of cell type portions was transformed using an isometric log-ratio (*ilr*) transformation (7–9). The transformation can be applied as long as all components are non-zero and leads to a (N-1) dimensional vector where the components are orthonormal and can be defined as:

$$z_{i}=\sqrt{\frac{N-i}{N-i+1}}log(\frac{x_{i}}{\sqrt[N-i]{\prod_{j=i+1}^{N} x_{j}}})$$

The first component $z_{1}$ remains interpretable in terms of the reference cell type chosen for $x_{1}$.

**Association between BP and placental cell-type composition**.

We first examined the possible influence of each maternal BP indicator on the *global* cellular composition through a multilinear regression model, adjusted for the following potential confounders: recruitment center, maternal age, child sex, maternal education level, BMI before pregnancy, parity, maternal active smoking; and technical factors. Association was tested for using an ANOVA (9):

$${ilr(Composition)}_{i}= 1 + \gamma{BP}_{i} + \gamma_{Z}^{T}Z_{i} + \varepsilon_{i}$$

Where ${BP}_{i}$ is the average BP level for participant *i* and $Z_{i}$ the set of adjustment factors.

In order to test for association between a continuous variable and a specific part of a composition in a linear regression setting, Hron e*t al.* suggested a model where the composition, considered as a dependent variable, is first transformed into linearly independent coefficients using the isometric-*logratio* transformation. By permuting the part chosen for $z_{1}$, one is thus able to test the association between a real-valued response and each part at a time while controlling for all other parts of the composition. We took advantage of this approach by swapping the roles of BP (now the independent variable) and cell-type composition. Association between each BP indicator and each reference cell-type was hence tested by fitting the following linear regression model:

$${BP}_{i} = \gamma_{0} + \gamma_{1}z_{1i} + \gamma_{\boldsymbol{2}}^{\boldsymbol{T}}\boldsymbol{(}z_{2i},z_{3i},z_{4i},z_{5i}) + \gamma_{Z}^{T}Z_{i}+\varepsilon_{i}$$

where the parameter $\gamma_{1}$ remains interpretable in terms of the relation between BP and the reference cell-type chosen for $z_{1}$(8)*.* The association between each BP indicator and the ratio of Stromal and Syncytiotrophoblast cells was evaluated using the following linear regression model, adjusted for the same confounders ($Z)$ as listed above:

$${log(Stromal/Syncytiotrophoblast)}_{i}= \gamma_{0}+ \gamma_{1}{BP}_{i} + \gamma_{Z}^{T}Z_{i} + \varepsilon_{i}$$

**Results**

**Supplementary Figure 3. Above: reference placental cell-type distributions observed in the EDEN cohort. Below: reference placental cell-type composition observed for each sample.**

**Analysis of Differentially Methylated Regions (DMRs)**

**Supplementary Figure 4 Pearson correlation between DMR effect sizes.** DMR effect sizes were computed as the average effect size of the included CpGs estimated in the EWAS.


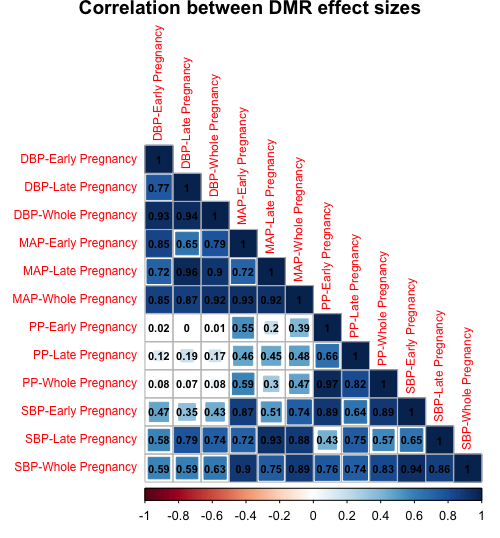


**Supplementary Figure 5A. Sensitivity analyses for DMRs found in our main analysis.** For each DMR detected in the main analysis**,** we compared the average effect sizes obtained in sensitivity analyses - adjusting for gestational duration (GA), excluding PE cases, adjusting for cell-type composition (CC) - to those computed in the main analysis. Median effect size log-ratios and the inter-quartile ranges are displayed. The dashed line indicates a ratio of 0.5. Average effect sizes estimated in the three sensitivity analyses are smaller than those obtained with our main model. However, they are only slightly discrepant from the main models when adjusting for gestational duration or excluding PE cases, whereas with observed marked differences (ratio<0.5) when models are adjusted for cell-type composition.


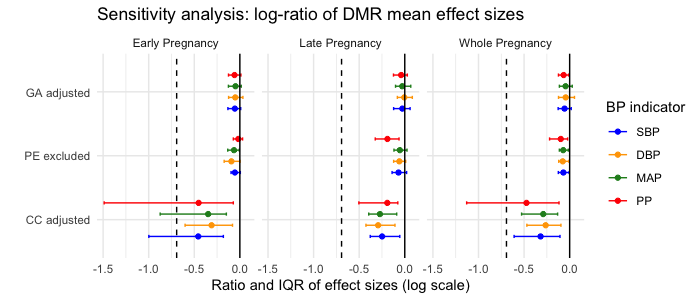


**Supplementary Figure 5.B. Sensitivity analysis excluding preeclampsia cases for the DMRs found in our main analysis.** We examined whether DMRs were still detected after excluding the 17 preeclamptic mothers (PE). DMRs detected in our main analysis, including PE cases (columns topped with white boxes) can be compared to DMRs detected after excluding PE cases (matched columns topped with black boxes). Numbers of DMR (including intergenic ones) that are conserved, lost or gained after excluding PE cases, are summarized in supplementary table ST8.A1.

**
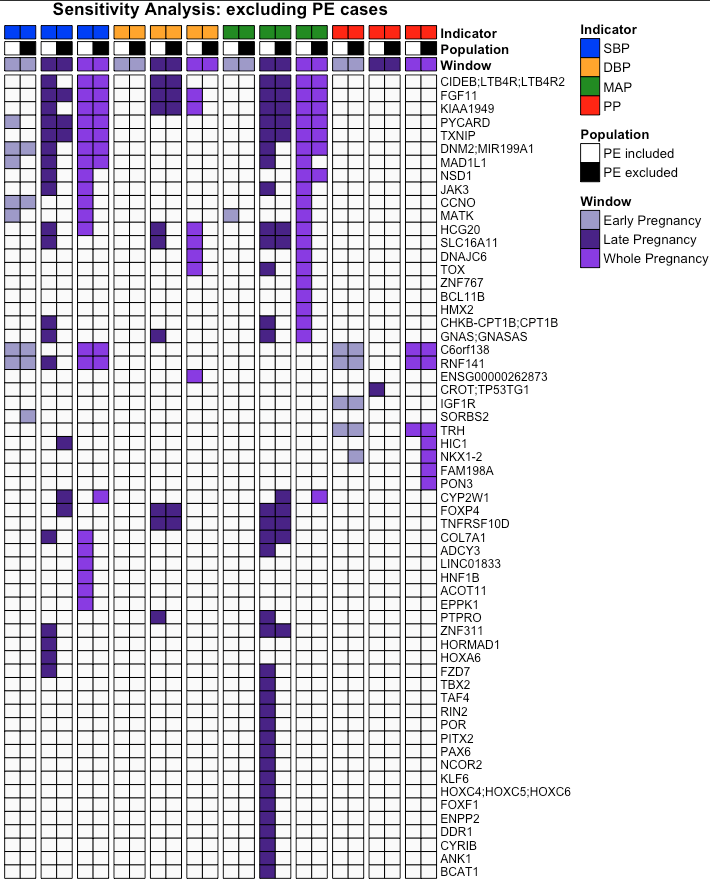
**

**Association between BP indicators and cell-type composition**

**Supplementary Figure 6.A. Magnitude and 95% confidence intervals of the association between each BP indicator and each reference cell-type (), estimated using the following linear regression framework:**

where the are the coordinates obtained after *ilr*-transformation of the cell-type mixture.

The portion of Stromal tissue is significantly associated (<0.05) with all four BP indicators over the three considered time windows (early, late and the whole pregnancy). The part of Syncytiotrophoblast significantly associated with SBP and MAP for all time windows and with DBP during the whole pregnancy and late pregnancy. See also Supplementary tables file. Two additional sensitivity analyses were performed, one by excluding preeclamptic mothers (PE), the other one by further adjusting the models for gestational age (GA).

**
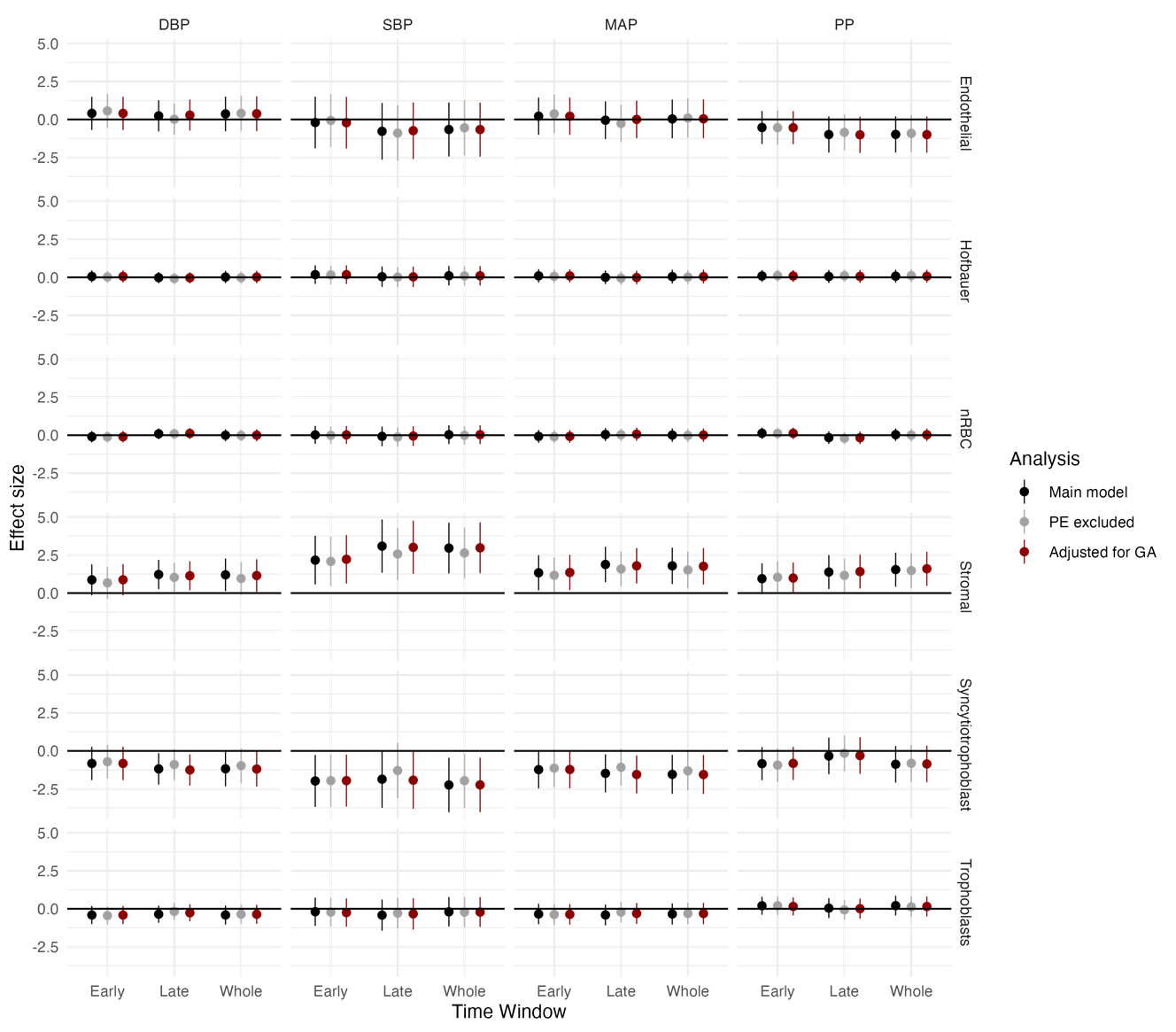
**

**Supplementary Figure 6.B. Compositional Principal Component Analysis performed on cell-type mixtures.** The correlation plot on the right hand shows that the Stromal and Syncytiotrophoblast parts tend to be correlated.

**Mediation Analysis**

**Supplementary Figure 7.A Diagnostic of the direct (DE) and indirect (IDE) effect (mediated by cell-type composition) of BP on the methylation level of DMRs.** DE and IDE were estimated CpG-wise using the R package *ccmm* and aggregated by DMR, thus defining three types of DMRs in both cases: those for which all CpGs have a significant effect (“All”), those for which no CpG has a significant effect (“None”) and those for which only a subset of CpGs have a significant effect (“Some”). Significance was determined from 95% bootstrap confidence intervals of the effect size.

**Supplementary Figure 7.B. Total, direct and indirect effect of BP on the methylation level of DMRs.** Total effects represented herein were broken up as the sum of the direct and indirect effects mediated by the ratio Stromal/Syncytiotrophoblasts. Effect magnitudes were computed as the absolute value of the mean effect size estimated for the CpGs included in the DMR, direct and indirect effects on a DMR were considered significant when it contained at least one probe for which the effect was found significant (p-value<0.05). SCT: Syncytiotrophoblast. Displayed DMRs are from the union of regions detected with regression models adjusted or/and not adjusted for cell-type composition.

**References**

1. Yang AS, Estécio MRH, Doshi K, Kondo Y, Tajara EH, Issa JJ. A simple method for estimating global DNA methylation using bisulfite PCR of repetitive DNA elements. Nucleic Acids Research. 2004 Feb 1;32(3):e38.

2. Zhao N, Bell DA, Maity A, Staicu AM, Joubert BR, London SJ, et al. Global Analysis of Methylation Profiles From High Resolution CpG Data. Genetic Epidemiology. 2015 Feb 1;39(2):53–64.

3. Kruppa J, Sieg M, Richter G, Pohrt A. Estimands in epigenome-wide association studies. Clinical Epigenetics. 2021 Apr 29;13(1):98.

4. Xiong Z, Li M, Yang F, Ma Y, Sang J, Li R, et al. EWAS Data Hub: a resource of DNA methylation array data and metadata. Nucleic Acids Research. 2020 Jan 8;48(D1):D890–5.

5. Kazmi N, Sharp GC, Reese SE, Vehmeijer FO, Lahti J, Page CM, et al. Hypertensive Disorders of Pregnancy and DNA Methylation in Newborns. Hypertension. 2019 Aug 1;74(2):375–83.

6. Phipson B, Maksimovic J, Oshlack A. missMethyl: an R package for analyzing data from Illumina’s HumanMethylation450 platform. Bioinformatics. 2016 Jan 15;32(2):286–8.

7. Teschendorff AE, Breeze CE, Zheng SC, Beck S. A comparison of reference-based algorithms for correcting cell-type heterogeneity in Epigenome-Wide Association Studies. BMC Bioinformatics. 2017 Feb 13;18(1):105.

8. Hron K, Templ M, Filzmoser P. Imputation of missing values for compositional data using classical and robust methods. Computational Statistics & Data Analysis. 2010 Dec 1;54(12):3095–107.

9. Templ M, Hron K, Filzmoser P. robCompositions: An R-package for Robust Statistical Analysis of Compositional Data. In: Compositional Data Analysis [Internet]. John Wiley & Sons, Ltd; 2011 [cited 2021 Oct 22]. p. 341–55. Available from: https://onlinelibrary.wiley.com/doi/abs/10.1002/9781119976462.ch25

10. Barton SJ, Melton PE, Titcombe P, Murray R, Rauschert S, Lillycrop KA, et al. In Epigenomic Studies, Including Cell-Type Adjustments in Regression Models Can Introduce Multicollinearity, Resulting in Apparent Reversal of Direction of Association. Frontiers in Genetics. 2019;10:816.

11. Hron K, Filzmoser P, Thompson K. Linear regression with compositional explanatory variables. Journal of Applied Statistics. 2012 May 1;39(5):1115–28.

12. van den Boogaart KG, Tolosana-Delgado R. Linear Models for Compositions. In: van den Boogaart KG, Tolosana-Delgado R, editors. Analyzing Compositional Data with R [Internet]. Berlin, Heidelberg: Springer; 2013 [cited 2021 Oct 28]. p. 95–175. (Use R!). Available from: https://doi.org/10.1007/978-3-642-36809-7_5

13. van den Boogaart KG, Filzmoser P, Hron K, Templ M, Tolosana-Delgado R. Classical and Robust Regression Analysis with Compositional Data. Math Geosci. 2021 Jul 1;53(5):823–58.
